# Supplementary material for: Functional and Stress Response Analysis of Heat Shock Proteins 40 and 90 of Giant River Prawn (Macrobrachium rosenbergii) under Temperature and Pathogenic Bacterial Exposure Stimuli
Source: Biomolecules. 2021 Jul 15;11(7):1034. doi: 10.3390/biom11071034 (PMC8301959; doi:10.3390/biom11071034)
Supplement: Supplementary file 1 [file biomolecules-11-01034-s001.zip › biomolecules-1275227-supplementary.pdf]

Scientific name	Common name	Accession number	Nucleotide identity (%)	Amio acid identity (%)	Similarity (%)
**Eukaryotic**					
**Vertebrate**					
Homo sapiens (*hsp40* type I)	Human	NM_001539.2	64.7	66.8	83.9
Homo sapiens (*hsp40* type II)	Human	NM_012266.5	48.1	32.3	49.2
Homo sapiens (*hsp40* type III)	Human	NP_001012339.2	45.2	20.4	36
Bos taurus (*hsp40* type I)	Cattle	NM_001015637.1	65.5	66.3	82.9
Mus musculus (*hsp40* type I)	Mouse	NM_008298.5	65.4	66.5	83.9
Gallus gallus (*hsp40* type I)	Chicken	NM_001012945.1	64.2	65.7	83.6
Anolis carolinensis (*hsp40* type I)	Carolina anole	XP_003224682.1	65	65.9	82.6
Xenopus tropicalis (*hsp40* type I)	Tropical clawed frog	NM_001011012.1	64.3	67	82.8
Danio rerio (*hsp40* type I)	Zebrafish	NM_199662.1	62.7	58.4	77.4
Danio rerio (*hsp40* type II)	Zebrafish	NP_001003571.1	46.6	29.3	48
Danio rerio (*hsp40* type III)	Zebrafish	NP_956338.1	45.5	19.5	35.8
**Invertebrate**					
Marsupenaeus japonicus	Japanese tiger prawn	AB520825.1	77.9	87.4	95.2
Paracyclopina nana	Copepod	HQ115579.1	55.8	54.5	68.9
Lepeophtheirus salmonis	Salmon louse	BT077397.1	60.7	56.5	73.5
Caligus clemensi	Sea lice	BT080459.1	54.7	48.9	68.6
Lottia gigantea	Owl limpet	XP_009066210.1	63	63.3	78.2
Spodoptera frugiperda	Fall armyworm	KF562156.1	65.5	69.3	82.5
Culex quinquefasciatus	Southern house mosquito	XM_001844740.1	61.2	62.6	77.7
Apis mellifera	Western honey bee	XM_006565940.1	62	63.1	79.9
Bombyx mori	Domesticated silkmoth	NM_001046827.1	63.5	67.9	82.3
Aedes aegypti	Yellow head mosquito	DQ440244.1	62.3	61.8	78.9
Tribolium castaneum	Red flour beetle	XM_966353.2	63.6	68.1	83.1
Caenorhabditis briggsae	Nematode	XM_002640654.1	57.2	48.5	66.3
**Plant**					
Arabidopsis thaliana	Arabidopsis	NM_122127.2	54.9	44.8	62.8
Chlamydomonas reinhardtii	Green algae	XM_001691546.1	51.9	42.4	59.6
Oryza sativa Japonica	Rice	NM_001058032.1	56.4	48.3	64.3
**Fungi**					
Schizosaccharomyces pombe 972h	Fission yeast	NM_001021336.1	54.5	47.4	64.9
Saccharomyces cerevisiae S288c	Yeast	NM_001182902.1	55.6	46	64.5
Magnaporthe oryzae	Rice blast fungus	XM_003710951_1	51.9	41.6	59.2
**Protozoa**					
Babesia bovis	Babesia	XM_001610861_1	51.3	39.2	58.5
Theileria parva strain Muguga	Theileria	XM_761134.1	50.8	37.7	57.7
Eimeria tenella	Eimeria	HG675727.1	52.1	43.4	63.4
**Prokaryotic**					
Pedobacter saltans DSM 12145	Pedobacter	NC_015177.1	49.8	34.6	55.6
Bacillus subtilis	Bacillus	AAA22529	50.4	30.6	51.5
Escherichia coli K12	E. coli	M12565	48.7	33	52.8

Supplemental material Table S1. Ju-Ngam *et al*. (2021)

Scientific name	Common name	Accession number	Nucleotide identity (%)	Amio acid identity (%)	Similarity (%)
**Eukaryotic**					
**Vertebrate**					
Homo sapiens (*hsp90*-alpha isoform 2)	Human	NM_005348.3	72.7	81.9	92.1
Homo sapiens (*hsp90*-alpha isoform 1)	Human	NM_001017963.2	62.5	67	75.5
Homo sapiens (*hsp90*-beta)	Human	NM_007355.2	72.8	80.9	90.7
Bos taurus (*hsp90*-alpha)	Cattle	NM_001012670.2	74.2	81.6	92.1
Mus musculus (*hsp90*-beta)	Mouse	NM_008302.3	74.2	80.9	90.9
Mus musculus (*hsp90*-alpha)	Mouse	NM_34610.1	73	81.3	92.3
Gallus gallus	Chicken	NM_001109785.1	74	81.5	91.7
Python bivittatus	Burmese python	XP_007437399.1	72.9	81.7	91.6
Xenopus tropicalis	Tropical clawed frog	NM_001016282.2	71.3	80.9	91.6
Danio rerio	Zebrafish	NM_131328.1	72.1	78.1	90.1
**Invertebrate**					
Apis mellifera	European honey bee	NM_001160064.1	70.8	84.6	91.9
Chlamys farreri	Japanese scallop	AY362761.1	70.8	79.3	89.9
Crassostrea gigas	Pacific oyster	EF687776.1	72.4	80.4	90.6
Haliotis asinina	Ass's-ear abalone	EF621884.1	75	81.5	92
Penaeus monodon	Giant tiger prawn	EF015590.1	80.7	90.3	96.3
Eriocheir sinensis	Chinese mitten crab	EU809924.1	82.8	90.9	96
Macrobrachium nipponense	Oriental River Prawn	GU319963.1	85.8	90.9	96.4
Scylla paramamosain	Green Mud Crab	JF265066.1	82.2	90.4	95.7
Marsupenaeus japonicus	Japanese tiger prawn	AB520827.1	81.5	90.5	96.3
Litopenaeus vannamei	Pacific white shrimp	HQ008268.1	80.9	90.2	95.7
Portunus trituberculatus	Swimming crab	FJ392027	82.1	90.6	95.9
Fenneropenaeus chinensis	Chinese shrimp	EF032650	79.6	89.1	96.3
Exopalaemon carinicauda	Ridgetail white prawn	HQ162267	76	81.4	91.1
Metapenaeus ensis	Sand shrimp	EF470247	81.1	90.6	96.3
Macrobrachium rosenbergii	Giant freshwater prawn	HG001457	75.9	80.9	90.1
**Plant**					
Arabidopsis thaliana	Arabidopsis	NM_124642.3	67.4	68.6	84.3
Triticum aestivum	Common wheat	ADF31760.1	69.6	68.3	83.7
Oryza sativa Japonica	Rice	NM_001070038.1	67.4	68.6	84.3
**Fungi**					
Lichtheimia corymbifera	Saprotrophic fungi	CDH57487.1	68.4	65.7	83.1
Coprinopsis cinerea	Mushroom	XP_001833710.1	67.7	63.2	80.3
Blastocladiella emersonii	Blastocladiella	ABH04243	68.5	66.1	82.9
**Protozoa**					
Eimeria tenella	Eimeria	CDJ41370.1	68	63.5	82
Plasmodium falciparum 3D7	Plasmodium	XM_001348962.1	60.3	62.5	77.4
Theileria parva	Theileria	XM_759717.1	64.8	64.2	80.7
**Prokaryotic**					
Escherichia coli strain TW14359	E. coli	EU904949	50.6	34.8	58.3
Myxococcus xanthus DK 1622	Myxococcus	YP_634186.1	53.5	38.7	60.1
Corallococcus coralloides DSM 2259	Myxobacteria	YP_005372538.1	53.3	39.5	60.6

Supplemental material Table S2. Ju-Ngam *et al*. (2021)
